# Transcriptome Kinetics of *Saccharomyces cerevisiae* in Response to Viral Killer Toxin K1

**DOI:** 10.3389/fmicb.2019.01102

**Published:** 2019-05-16

**Authors:** Stefanie Gier, Martin Simon, Karl Nordström, Salem Khalifa, Marcel H. Schulz, Manfred J. Schmitt, Frank Breinig

**Affiliations:** ^1^Department of Molecular and Cell Biology, Saarland University, Saarbrücken, Germany; ^2^Center of Human and Molecular Biology (ZHMB), Saarland University, Saarbrücken, Germany; ^3^Molecular Cell Biology and Microbiology, University of Wuppertal, Wuppertal, Germany; ^4^Molecular Cell Dynamics, Saarland University, Saarbrücken, Germany; ^5^Department of Genetics/Epigenetics, Saarland University, Saarbrücken, Germany; ^6^Cluster of Excellence “Multimodal Computing and Interaction”, Max Planck Institute for Informatics, Saarland University, Saarbrücken, Germany

**Keywords:** K1, killer toxin, yeast viral toxin, *Saccharomyces cerevisiae*, transcriptome

## Abstract

The K1 A/B toxin secreted by virus-infected *Saccharomyces cerevisiae* strains kills sensitive cells via disturbance of cytoplasmic membrane functions. Despite decades of research, the mechanisms underlying K1 toxicity and immunity have not been elucidated yet. In a novel approach, this study aimed to characterize transcriptome changes in K1-treated sensitive yeast cells in a time-dependent manner. Global transcriptional profiling revealed substantial cellular adaptations in target cells resulting in 1,189 differentially expressed genes in total. Killer toxin K1 induced oxidative, cell wall and hyperosmotic stress responses as well as rapid down-regulation of transcription and translation. Essential pathways regulating energy metabolism were also significantly affected by the toxin. Remarkably, a futile cycle of the osmolytes trehalose and glycogen was identified probably representing a critical feature of K1 intoxication. *In silico* analysis suggested several transcription factors involved in toxin-triggered signal transduction. The identified transcriptome changes provide valuable hints to illuminate the still unknown molecular events leading to K1 toxicity and immunity implicating an evolutionarily conserved response at least initially counteracting ionophoric toxin action.

## Introduction

The killer phenomenon in yeast relies on the secretion of proteinaceous compounds, so-called killer toxins, with lethal effects on sensitive yeast cells of the same or other genera. In case of the baker’s yeast *Saccharomyces cerevisiae*, killer strains are infected with two double-stranded RNA viruses, referred to as M and L-A, that are persistently present in the cytoplasm as virus-like particles (VLPs). To date, four different killer toxins (K1, K2, K28, and Klus) have been identified for *S. cerevisiae* based on their killing properties and the lack of cross-immunity, which are all expressed as precursor proteins undergoing further maturation in the secretory pathway of the host cell (Wickner et al., 2013). Among these, K1 shares several features with medically relevant ionophoric A/B toxins making the toxin a valuable tool to study its effects in the well-established eukaryotic model organism *S. cerevisiae*.

Despite the lack of sequence similarity and different modes of action, both structural composition and processing of K1 and K28 precursor molecules within the secretory pathway are similar (Bostian et al., 1984; Riffer et al., 2002). Besides an N-terminal signal peptide (SP) necessary for the import into the lumen of the endoplasmic reticulum (ER), the unprocessed precursor consists of two major toxin subunits α and β, separated by a γ component probably acting as an internal chaperone during passage through the secretory pathway. After cleavage of the signal peptide in the ER and potential N-glycosylation of the γ subunit, α and β are covalently linked via one or more disulfide bonds, and the toxin undergoes additional processing steps in the late Golgi compartment involving the peptidases Kex2p and Kex1p. Eventually, the mature heterodimeric A/B toxin is secreted into the extracellular space (Schmitt and Tipper, 1995).

*Saccharomyces cerevisiae* virus toxins kill sensitive yeast cells in a two-staged receptor-mediated process, in which the first step involves a rapid and energy-independent binding to the respective primary receptor within the cell wall (Hutchins and Bussey, 1983; Schmitt and Radler, 1988). After transfer to the plasma membrane, an energy-dependent interaction with individual secondary receptors initializes the lethal effect of each killer toxin. K28 is parasitizing the retrograde transport pathway of the target cell by binding to Erd2p, finally reaching the nucleus where the toxic α subunit induces blockage of DNA synthesis resulting in cell cycle arrest (Eisfeld et al., 2000; Becker et al., 2016). K1 killer toxin, in contrast, binds to Kre1p, an *O*-glycosylated and transiently GPI-anchored protein involved in cell wall composition via β-1,6-glucan biosynthesis (Breinig et al., 2002, 2004).

Investigation of K1 structure, synthesis, and secretion has revealed valuable insights into essential cell biological mechanisms underlying the processing of proteins in the secretory pathway (Schmitt and Breinig, 2002). Its toxic effect was first described as alterations in plasma membrane permeability of sensitive yeast and leakage of ATP molecules (Bussey and Sherman, 1973). Additionally, inhibition of net proton pumping, amino acid/proton co-transport, and uncontrolled efflux of potassium ions was observed after K1 toxin addition (Skipper and Bussey, 1977; de la Pena et al., 1980). Whereas the latter was attributed to a compensatory effect of the proton influx into the cytoplasm, the monitored stop of proton pumping was interpreted as an artifact of the substantial toxin-induced H^+^ influx that cannot be compensated by the plasma membrane ATPase, by now known as Pma1p (de la Pena et al., 1981). However, despite many efforts to explore the exact molecular mechanisms and the role of potential accessory proteins, the mechanism underlying K1 toxicity could not be clarified yet. The current model of toxin action allows both, a direct insertion into the membrane as well as further interaction with one or more so far unknown primary effector protein/s (Breinig et al., 2002). Although the toxic mechanism of K2 is not entirely investigated yet, several studies suggested a killing mechanism comparable to K1 which is cumulating in disruption of the plasma membrane integrity (Meskauskas and Citavicius, 1992; Magliani et al., 1997).

In contrast to bacterial toxin producers lacking the particular toxin-specific eukaryotic target, killer yeast cells require particular self-immunity mechanisms against their own toxin. In case of K28, toxin precursor molecules in the cytoplasm of the killer cell bind to re-internalized mature toxin giving rise to proteasomal degradation of the whole complex (Breinig et al., 2006). Immunity against K1 was proposed to result from a loss of its secondary receptor Kre1p from the plasma membrane of killer cells due to interaction with the protoxin during the maturation process cumulating in vacuolar degradation of the Kre1p/proK1 complex (Sturley et al., 1986; Tipper and Schmitt, 1991). However, in recent experiments using intracellularly expressed K1 derivatives in a Δ*kre1* background, we were able to show that Kre1p is not involved in processes confirming immunity to K1 killer yeast. Nevertheless, expression of the preprotoxin, but not a derivative lacking the ER-import signal, in a sensitive cell grants immunity to externally applied K1, clearly pointing to a mechanism involving proteins of the secretory pathway and the precursor itself (Gier et al., 2017). Similar to the toxic effect, the exact mechanism leading to immunity in K1 killer cells and the involved molecules need further investigation.

In order to gain insight into the K1 toxic mechanism and the possibilities of a sensitive yeast to resist its toxic effect, we analyzed the transcriptional response of K1-treated *S. cerevisiae*. Therefore, we conducted a time-series experiment to initially dissect the kinetics of early, intermediate, and late responding genes. Our results identify a variety of cellular pathways with differentially expressed genes indicating a massive but dynamic cellular response. These data will be useful to establish a transcriptome resource for future validation of identified genes using biochemical approaches addressing not only K1 biology in particular but also medically relevant ionophoric A/B toxins in general.

## Materials and Methods

### Strains and Culture Media

The K1-sensitive *S. cerevisiae* strain BY4742 (MATα *his3*Δ*1 leu2*Δ*0 lys2*Δ*0 ura3*Δ*0*) was obtained from DharmaconGE. K1 toxin was produced by *S. cerevisiae* strain T158c (MATα his4C-864). Cells were grown at 30°C in YPD (1% yeast extract, 2% peptone, and 2% glucose). All deletion mutants used in this study were obtained from Euroscarf (Frankfurt) with a BY4742 background except for the essential gene *PMA1* which was tested in a diploid background (BY4743, MATa/MATα; *ura3*Δ*0*/*ura3*Δ*0*; *leu2*Δ*0*/*leu2*Δ*0*; *his3*Δ*1*/*his3*Δ*1*; *met15*Δ*0*/MET15; LYS2/*lys2*Δ*0*, YGL008c/YGL008c::kanMX4).

### Toxin Production and Concentration

T158c was cultivated in complex JG medium [0.75% yeast extract, 0.5% peptone, 0.5 × 10^-3^% FeCl_3_, 0.5 × 10^-3^% MnSO_4_, 0.5% MgSO_4_, 0.4% (NH_4_)_2_SO_4_, 0.08% KCl, 1.0% glucose, 5.2% glycerol] adjusted to pH 4.7 with McIlvaine buffer (Flegelova et al., 2002) (adapted). After cultivation for 5 days at 20°C, yeast cells were harvested via centrifugation, and the resulting cell-free supernatant was concentrated by an ultrafiltration device (Amicon, Sartorius) using appropriate ultrafiltration discs (PLCC07610, Merck-Millipore) with a molecular cut-off of 5 kDa. Finally, the toxin concentrate was dialyzed against 10 mM McIlvaine buffer (pH 4.7) over 3 days.

### *In vivo* Toxicity Assay

Toxicity of K1 samples and screening of the deletion mutants was determined with a standard agar diffusion assay using methylene blue agar (MBA, pH 4.7). After embedding 10^6^ cells per plate, 100 μl toxin concentrate was applicated into distinct pre-cut holes. Upon incubation for 3 days at 20°C, the diameter of the inhibition zone was measured and considered to reflect killer toxin activity further expressed in arbitrary units (AU). 1,000 AU correlate to a zone of growth inhibition of 10 mm in diameter against strain *S. cerevisiae* BY4742. Killing zone of the tested deletion mutants was normalized to wild-type killing zone (*n* = 5). Hypersensitivity was defined as the formation of killing zones with a 120% diameter or higher compared to the wild-type.

### Experimental Design for Transcriptome Analysis

Sensitive yeast cells of *S. cerevisiae* BY4742 were cultivated in JG medium at 20°C and 110 rpm over several days including subcultivation to adapt the cells metabolically to the experimental conditions. For RNA isolation, the main culture was inoculated to a start OD_600_ of 0.1 and incubated to a final OD_600_ of 0.5 (110 rpm, 20°C). Three times 10 ml of the suspension were harvested (1,200 rpm, 3 min, 4°C) and the resulting cell pellet was snap-frozen in liquid nitrogen, resulting in three independent technical replicates. The remaining culture was incubated with K1 killer toxin with an end concentration of 1,000 AU. Samples were taken at different time points (2, 10, 30, 60, and 120 min) and handled as mentioned above. Snap-frozen pellets were covered with 1 ml Tri-Reagent (Sigma), placed on dry ice, and subsequently transferred into appropriate tubes. Cell disruption was performed using glass beads and a cell homogenizator, samples were centrifugated (14,000 rpm, 1 min, RT), and subsequently used for RNA isolation.

### RNA Isolation and Illumina Sequencing

Total RNA was extracted using the Direct-zol^TM^ RNA isolation Kit (Zymo Research) including on-column DNase I digestion. Integrity of RNA was verified, and concentration was measured via Qubit^TM^ RNA HS Assay Kit (Invitrogen). Poly-A enrichment from 1 μg total RNA was performed via mRNA isolation method [NEBNext^®^ Poly(A) mRNA Magnetic Isolation Module]. Library preparation was carried out using the NEBNext^®^ Ultra^TM^ II DNA Library Prep Kit for Illumina^®^ using 15 min for cDNA fragmentation and 8 PCR cycles. After size selection with AMPure Beads, the library’s content was checked for adapter content and size distribution using the Agilent Bioanalyzer and the Agilent High Sensitivity DNA Kit. Libraries were multiplex sequenced on an Illumina HiSeq2500 platform using High Output mode with 1 × 100 nt read length. Reads were demultiplexed with bcl2fastq (v1.8.4). Adaptor trimming for adaptor contamination of the Illumina TruSeq adapter AGATCGGAAGAGC and quality trimming of low quality 3′ read end (*Q* ≤ 20) were performed with the cutadapt wrapper (v1.4.1), and adaptor sequences were trimmed with TrimGalore (v0.3.3) (Martin, 2011). Raw data were deposited at the European Nucleotide Archive (ENA) with the study accession number PRJEB28672^1^. FASTq files are labeled as follows BY4742_K1 (Control replicate 1), BY4742_K2 (Control replicate 2), BY4742_303 (t_30_ replicate 3), etc.

### Analysis and Visualization of Transcriptome Data

Each replicate dataset was used for gene expression quantification using Salmon (v0.8.2) (Patro et al., 2017). The information about reads per sample and reads used for quantification are displayed in Supplementary Table S1. Gene expression counts and TPM values were computed using the command: salmon quant -i transcriptIndex -l IU -1 reads1.fq -2 reads2.fq. The index for quantification with the software was constructed from all Ensembl cDNA sequences obtained in the downloaded file Saccharomyces_cerevisiae.R64-1-1.cdna.all.fa.gz (Zerbino et al., 2018). For all comparisons in the paper, except DESeq2 analysis, TPM counts were used to normalize the differences in sequencing depth.

The DESeq2 method (v1.18.1) was used to determine differentially expressed genes (DEGs) using gene expression read counts obtained by Salmon as DESeq applies its own normalization. Further, multiple testing corrected (adjusted) *p*-values < 0.01 (Love et al., 2014) were used as thresholds. Up- and down-regulated DEGs obtained above were divided and GO enrichment using the ‘Gene Ontology enRIchment anaLysis and visuaLizAtion tool’ (GOrilla) was conducted (Eden et al., 2009). Comparison was performed by inserting the respective DEG set and the gene background set of *S. cerevisiae* S288C. The resulting list of GO terms was further shortened according to false discovery rate (FDR)-corrected *q*-value with a cut-off of 0.01 and used for subsequent reduction in REVIGO (‘REduce and VIsualize Gene Ontology’) allowing small (0.5) similarity and applying SimRel score as semantic measurement (Supek et al., 2011). For fold enrichment analysis the hierarchical clustering of REVIGO was used to determine the generic GO term; for simplification, a cut-off of a minimal fold enrichment of 3 was chosen. In case of GO term enrichment of time-dependent data, up- and down-regulated DEGs were initially further clustered according to the defined time points (EARLY, INTERMEDIATE, and LATE). GO analysis was conducted as mentioned above (FDR-*q* < 0.01). The resulting list of GO terms was used for reduction in REVIGO (small similarity, SimRel). Graphs were plotted using the calculated log10 *p*-value as a function of semantic space Y with sphere size and color corresponding to log10 *p*-value (blue: highest significance).

### DREM2.0 Analysis

The mean of each triplicate was calculated as an expression data input file for the ‘Dynamic Regulatory Events Miner’ (DREM) (Schulz et al., 2012), and ‘log normalize data’ was selected as a preprocessing option. The file ‘yeast_anycond001’ that comes with DREM was used as transcription factor (TF)-gene interaction template and the gene annotation source was set to ‘yeast’. Based on the observed log fold changes in the time-course data set, the minimal absolute expression change was set to 0.3. Additionally, Bonferroni correction was applied as multiple hypothesis correction method for the GO analysis within DREM 2.0. TF assignment at created split nodes was set to a threshold score of *p* < 0.01.

### Flow Cytometry and Determination of Cell Viability

Pore formation was determined by propidium iodide (PI) staining and subsequent FACS analysis. BY4742 cells were grown in YPD, subsequently shifted in JG medium, and K1 toxin was added to a final concentration of 1,000 AU. Samples were taken over the course of 5 h, washed in 1 × PBS, and fixed in 1% paraformaldehyde (in 1 × PBS). After the transfer to appropriate tubes, 1 μg/ml PI (stock solution 1 mg/ml in PBS, Sigma) was added, and cells were incubated for 10 min on ice. Analysis was performed using BD LSRFortessa Cell Analyzer; data were analyzed with BD FACSDiva^TM^ software (BD Bioscience, Heidelberg, Germany). Non-stained and heat-treated yeast cells were used for instrument settings; all samples were measured in triplicates with 100,000 gated events each. Samples for determination of cell viability were taken simultaneously, washed with 1 × PBS and plated on YPD agar plates considering appropriate dilutions and replicates.

### Determination of Intra- and Extracellular Metabolite Levels

BY4742 cells were cultivated in JG medium until OD_600_ 0.5 was reached and subsequently incubated with 1,000 AU K1 toxin concentrate. Samples were taken over 5 h, and directly quenched in 60% methanol (-40°C); extraction was performed as described by Sasidharan et al. (2012). Briefly, cell-free supernatant was collected after short centrifugation and lyophilized (extracellular). Intracellular metabolite extraction was performed via cell lysis using methanol-chloroform and glass beads. Samples were centrifuged, the aqueous layer was recovered (intracellular), and also lyophilized. ATP measurement was conducted using the luminescence-based “ATP Determination Kit” (Thermo Fisher), Glycerol levels were determined using the “Free Glycerol Assay Kit” (Abcam). All samples were prepared in triplicates and kits were used according to the instructions of the manufacturer.

## Results

**FIGURE 1:**
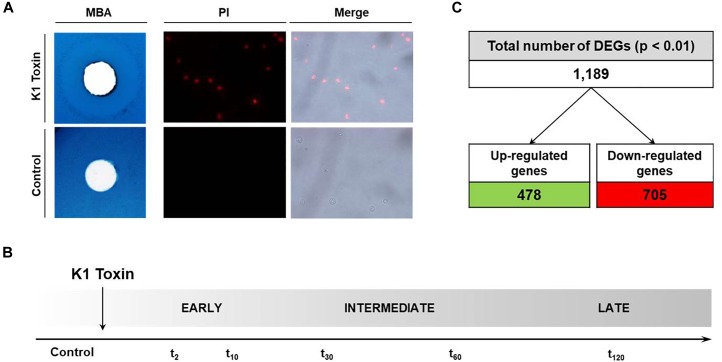
Transcriptional analysis of K1-treated sensitive cells. (**A**, left) Growth inhibition zone produced by 1,000 AU K1 toxin concentrate. 10^6^ cells of the sensitive yeast strain BY4742 were embedded in MBA (pH 4.7), and K1 toxin or heat-inactivated toxin (15 min, 100°C) were applicated into pre-cut wells. After incubation of 3 days at 20°C, the diameter of the growth inhibition zone was measured. (**A**, right) K1-mediated pore-formation in a sensitive cell. *S. cerevisiae* BY4742 cells were incubated with 1,000 AU K1 or McIlvaine buffer (pH 4.7, control) and stained with 1 μg/ml PI. Fluorescence was visualized using fluorescence microscopy (λ_em_ 617 nm). **(B)** Experimental set-up. To obtain a broad time-dependent overview of transcriptome changes in the sensitive yeast BY4742, samples for RNA isolation were prepared after different toxin incubation times. Control samples were taken before K1 application (final concentration of 1,000 AU). Sampling points were clustered into early (*t*_2_ and *t*_10_), intermediate (*t*_30_ and *t*_60_) and late (*t*_120_), respectively. **(C)** Number of DEGs over all time points. In total, 1,189 DEGs were obtained (adjusted *p* < 0.01), out of these 478 ORFs were up-regulated and 705 down-regulated in comparison to the untreated control samples; 6 DEGs showed fluctuating fold changes over time (not depicted).

In preliminary experiments, the properties of the used K1 concentrate were determined regarding time dependency of its toxic effect a well as appropriate concentration for efficient cell killing. A final concentration of 1,000 AU was chosen producing a 10 mm zone of growth inhibition against BY4742 in an MBA plate assay (Figure 1A, left panel). By applying a high concentration, we excluded the induction of apoptosis as it has been already observed for lower doses of K1 (Reiter et al., 2005). Additionally, a PI staining of samples incubated with the determined K1 amount was performed, indicating the start of a PI-detectable pore-formation at ca. 80 min (Figure 1A, right panel). Based on these observations, the experimental set-up for RNA isolation was scheduled with sampling points at 2, 10, 30, 60, and 120 min after toxin application (Figure 1B). The induced transcriptome adaptations were compared with the untreated control samples taken before toxin supplementation. We were able to identify 1,189 DEGs, which were significantly de-regulated (adjusted *p*-value < 0.01) by considering the union over all time windows assessed with DEseq2 (see section “Materials and Methods”). These include 478 up-regulated and 705 down-regulated ORFs (Figure 1C); only six genes (*MIG2*, *MUP1*, *CHO2*, *HSP82*, *SSA1*, and *GPP2*) showed fluctuating fold changes over time (complete list of DEGs in Supplementary Table S2).

### Global K1-Induced Transcriptome Alterations

**FIGURE 2:**
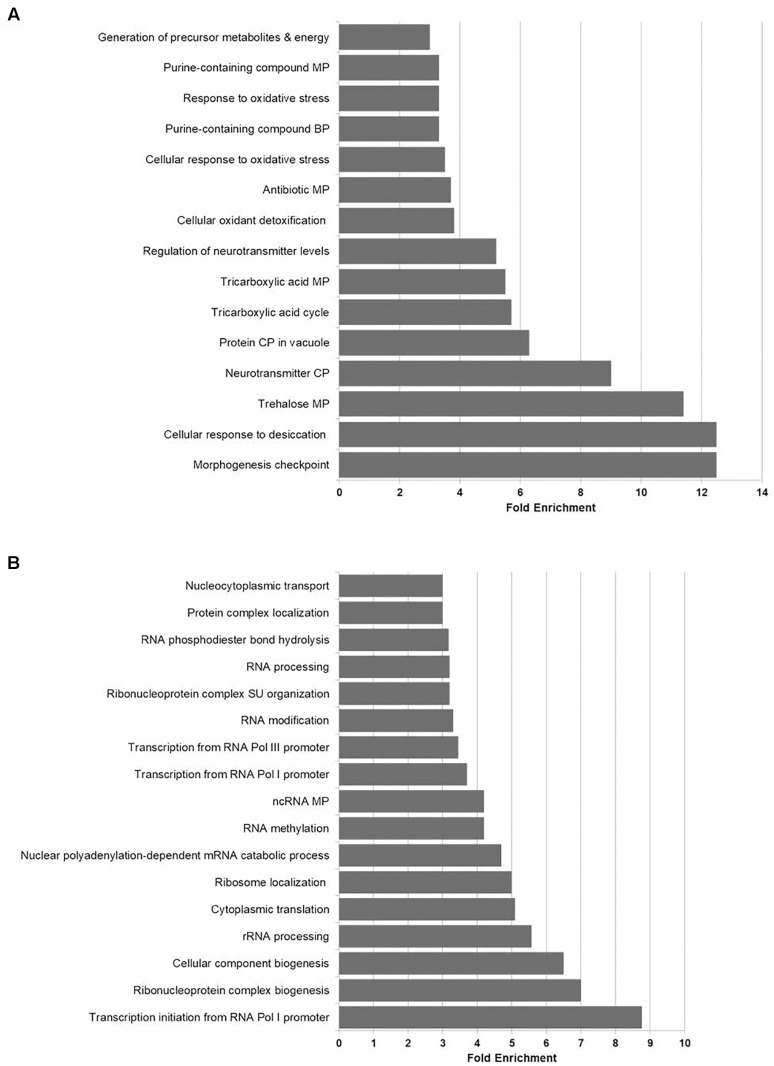
Fold enrichment of up-regulated **(A)** and down-regulated **(B)** GO terms associated with biological processes. DEGs were classified in up- and downregulated genes according to the corresponding log2 fold change (adjusted *p* < 0.01). Enrichment was performed using GOrilla software, subsequent clustering and reduction of the significant terms (FDR-corrected *q*-value < 0.01) were carried out via REVIGO. For simplification reasons, only GO terms with a fold enrichment >3 are depicted (complete list in Supplementary Table S3). BP, biological process; CP, catabolic process; MP, metabolic process; mRNA, messenger RNA; ncRNA, non-coding RNA; Pol, polymerase; rRNA, ribosomal RNA; SU, subunit.

In order to get a general overview on cellular activities affected by K1, data were initially analyzed by calculating a gene ontology (GO) term enrichment of ‘biological process,’ ‘molecular function,’ and ‘cellular compartment’ over all time points. Subsequent clustering and reduction of the significant terms were carried out using the REVIGO tool (complete lists of GO terms in Supplementary Table S3). In transcriptional response to extracellular K1 toxin application, several stress implications like ‘oxidative stress response’ and ‘trehalose metabolism’ were up-regulated with a fold enrichment of 3.5 and 11.4, respectively (Figure 2A). Also, the metabolic processes of ‘purine-containing compounds’ (3.3) and ‘tricarboxylic acid (TCA) cycle’ (5.5) were enriched in up-regulated DEGs. The highest fold enrichment could be observed for the biological processes ‘morphogenesis checkpoint’ and ‘cellular response to desiccation’ (12.5 each). Whereas the former is the description of a mitotic checkpoint regulating the delay of the cell cycle in response to perturbations affecting cell shape or size, the latter refers to genes connected to trehalose metabolism. Moreover, significant fold enrichment in energy metabolism regarding the ‘generation of precursor metabolites’ could be observed (3.0). In the case of GO analysis of down-regulated DEGs, we found several significantly enriched biological processes like regulation of gene expression, transcription and cytoplasmic translation (Figure 2B). Among these, ‘transcription from RNA polymerases I and III promoter’ were observed with fold enrichments of 3.7 and 3.5, respectively, including ‘transcription initiation from RNA Polymerase I promoter’ (8.8). DEGs from biological processes regarding ‘RNA modification,’ ‘RNA methylation’ as well as ‘RNA processing’ were also significantly down-regulated (fold enrichment of 3.2, 4.2, 3.2, respectively). Furthermore, biogenesis, assembly and localization of ribosomal subunits (SU) tend to be impaired by K1 toxin with fold changes of 3.2 (‘ribonucleoprotein complex SU organization’), 6.5 (‘cellular component biogenesis’) and 7.0 (‘ribonucleoprotein complex biogenesis’). The ‘molecular function’ and ‘cellular component’ GO enrichment confirmed these observations and emphasized the importance of trehalose metabolism suggesting a crucial role of the osmolyte in potential defense mechanisms against the ionophoric effect of K1 (see Supplementary Table S3).

### Time-Resolved Transcriptome Adaptations

**FIGURE 3:**
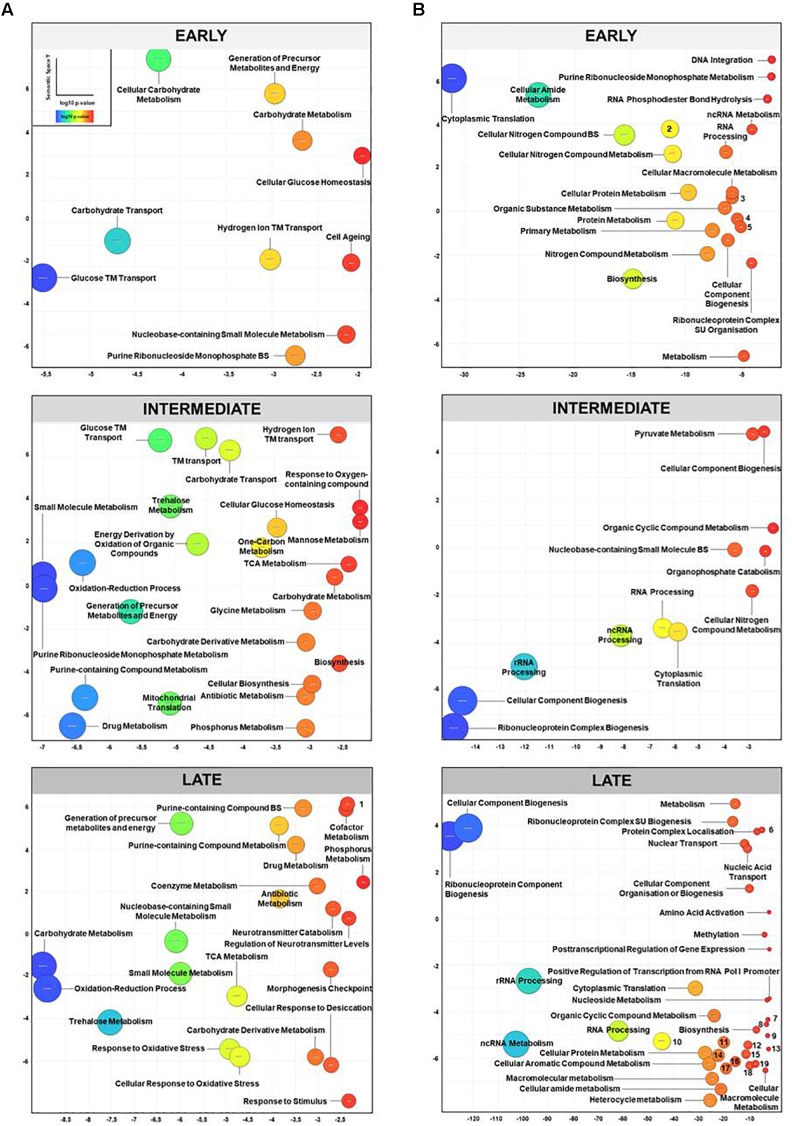
Temporal distribution of significantly de-regulated GO terms after K1 application. DEGs (*p* < 0.01) were clustered into early (*t*_2_ and *t*_10_), intermediate (*t*_30_ and *t*_60_) and late responding genes (*t*_120_) after K1 application and sorted according to their respective log2 fold change in up- or down-regulated genes. GO enrichment was performed using GOrilla software for each data set. The subsequent visualization of the significantly enriched GO terms (FDR-corrected *q*-value < 0.01) was performed via REVIGO. GO terms are plotted according to calculated log10 *p*-value (*X*-axis) and semantic space (*Y*-axis, implicating similarity between terms). Color and size of the spheres correlate with log10 *p*-value (blue: highest significance). **(A)** Up- and **(B)** down-regulated GO terms. For facilitated reading labeling of smallest spheres was replaced by numbers 1: Pyridine-containing compound metabolism, 2: Organonitrogen compound metabolism, 3: Ribonucleoprotein complex metabolism, 4: Cellular metabolism, 5: Cellular complex biogenesis, 6: Cellular process, 7: Transcription from RNA Polymerase (Pol) III promoter, 8: Transcription from RNA Pol I promoter; 9: Cellular macromolecule metabolism, 10: Cellular nitrogen compound metabolism, 11: Organic substance metabolism, 12: Cellular nitrogen compound biosynthesis, 13: Cellular macromolecular metabolism, 14: Primary metabolism; 15: RNA modification, 16: RNA phosphodiester bond hydrolysis, 17: Cellular metabolism: 18: Nucleic acid phosphodiester bond hydrolysis, 19: RNA methylation.

In order to analyze the temporal distribution of up- and down-regulated genes after toxin application, we categorized all significantly altered DEGs of both gene sets in early (2 and 10 min), intermediate (30 and 60 min) and late responders (120 min; see Figure 1B). For up-regulated ORFs, 99 genes were classified as early responders, 139 as intermediate and 404 as late responding genes, respectively. In contrast to this gradual increase in the amount of de-regulated genes over time, we found 200 ORFs to be down-regulated at early time points and 176 genes at intermediate sampling points. Interestingly, after 120 min of K1 toxin incubation, we observed a sharp increase in responding genes to 622 ORFs considered as late responders. GO term enrichment was performed with each set of genes using GOrilla, and the resulting list of GO terms was visualized via REVIGO. We found several terms enriched pointing to an up-regulation of biological processes to cope with the toxic effect of K1 (Figure 3A). In all stages, genes of the carbohydrate metabolism are highly up-regulated. Especially at early time points, many genes concerning hexose metabolism and ‘transmembrane transport’ were significantly enriched embracing amongst others *GSY1-* and *GSY2-*encoded glycogen synthases, the glucokinases-coding gene *GLK1*, and several high-affinity hexose transporters (Figure 3A, EARLY). The biological process ‘hydrogen ion transmembrane transport’ refers to genes coding for mitochondrial proteins (*ATP14*, *ATP15*, *COX8*, *PIC2*, *TIM11*, *etc.*) whereas ‘cell aging’ includes genes like *TEC1* and *MSN4* coding for TFs, the superoxide dismutase-encoding *SOD2*, and the thioredoxin peroxidase Tsa1p. The latter acts as an antioxidant in both mitochondria and cytoplasm and hints, together with the up-regulation of *SOD2*, to the first encounter of the cell with oxidative stress. The intermediate response to K1 comprises the already early up-regulated GO terms ‘carbohydrate metabolism’ and ‘trehalose metabolism’ also found to be highly enriched in the GO enrichment performed over all time points (see Figure 2A). ‘Trehalose metabolism’, also significantly up-regulated at late sampling points, includes the genes *TPS1*, *TPS2*, *PGM2*, *TSL1*, and *UGP1* (later also *ATH1*, *NTH1*, and *NTH2*) and, thus, seems to play an essential role in the stress response to the toxic effect of K1 (Figure 3A, INTERMEDIATE). We also found genes annotated with the process of ‘mitochondrial translation’ up-regulated covering 15 ORFs of mitochondrial proteins (e.g., *MNP1*, *MRPL23*, *MRPL13*, *MRPL1*, *MRPL3*, and *MRSP12*). Furthermore, ‘metabolism of purine-containing compounds’ was significantly enriched and up-regulated DEGs contained many genes of the ‘*de novo* IMP synthesis’ (e.g., *ADE1*, *ADE4*, *ADE12*, *ADE13*, and *ADE17*) which were already up-regulated at early time points. As a further intermediate response to K1 toxin application, with *IDH1*, *IDH2*, *LSC2*, *SDH3*, *SDH4*, and *CIT1*, 6 TCA cycle components were up-regulated. Additionally, the GO terms ‘drug metabolism’ and ‘antibiotic metabolism’ summarize many TCA cycle-related genes as well as oxidative stress responders.

The GO term analysis for late responding genes was similar to the intermediate responders (Figure 3A, LATE). However, we found some additional biological processes to be up-regulated which are related to several stress responses. These processes comprise the genes necessary for the trehalose metabolism, *TPS1*, *TPS2 NTH1*, *ATH1*, and *TSL1* (‘trehalose metabolism’ and ‘cellular response to desiccation’), for oxidative stress (*MXR2*, *GRX1*, *GRE3*, *CTT1*, *HYR1*, *PRX1*, and *ZTA1*), and genes for a more general cell stress response; the latter includes *XBP1*, *MSH2*, *MSH6*, and *DDR2* coding for multi-stress responsive proteins. GO terms ‘neurotransmitter catabolism’ and ‘regulation of neurotransmitter levels’ were also positively enriched. These cover the genes *GCV1*, *GCV2*, and *GCV3* coding for all three subunits of the mitochondrial glycine decarboxylase complex and additionally the glycine hydroxymethyltransferase-encoding gene *SHM2* catalyzing the conversion of serine to glycine. However, several heat shock proteins were up-regulated, with the highest fold changes for *HSP12, HSP26*, and *HSP42* (1.8, 1.4, and 1.2, respectively). The term ‘morphogenesis checkpoint’, that was already enriched in the general GO analysis was also found to be up-regulated in late stages of K1 toxin action, consisting of the genes *SWE1*, *GIN4*, *HSL1*, and *KCC4*, hinting to perturbations of cell shape and cell wall integrity.

In case of GO terms of down-regulated DEGs, many ORFs in the categories ‘cytoplasmic translation’ and ‘RNA processing’ were enriched immediately at early sampling points (Figure 3B, EARLY). Several genes of ribosomal structural constituents including most of the ORFs for both SU were down-regulated. Additionally, genes belonging to RNA processing (including ‘ncRNA and rRNA processing’) were negatively regulated at early time points. The term ‘DNA integration’ refers to 8 down-regulated retrotransposable genes. Interestingly, we found 24 Ty genes in total to be significantly de-regulated in comparison to the untreated control; over all time points, seven were up-regulated, whereas most were negatively regulated (see Supplementary Table S2). In contrast to the rather moderate fold changes observed in general, retrotransposons showed high transcriptional alterations with fold changes of approximately 6–10. GO term enrichment for intermediate down-regulated genes displayed a slight decrease in responding ORFs mirrored in the lower log10 *p*-values, whereas cellular ‘component biogenesis’ and ‘ribonucleoprotein complex’ referring to ribosome biogenesis and assembly were still highly significantly enriched. Actually, ‘rRNA and ncRNA processing’ were the GO terms with the next highest log10 *p*-values demonstrating the ongoing down-regulation of genes at intermediate sampling points after K1 application (Figure 3B, INTERMEDIATE). The transcriptional profiling of lately down-regulated DEGs revealed the highest abundance of GO terms concerning gene expression in general, transcription and translation. Ribosomal biosynthesis, arrangement, and assembly displayed in the terms ‘ribonucleoprotein component biogenesis’ and ‘cellular component biogenesis,’ showed the most significant enrichment in the whole analysis. ‘Regulation of transcription from RNA Polymerase (Pol) I respective III’ was additionally obtained (e.g., *RPC10*, *RPC19*, *RPA4*, and *RPB5*) as well as ‘cytoplasmic translation’ (Figure 3B, LATE).

### Regulation of Ion Transporters

Although the mechanism of K1 toxin action remains to be fully elucidated, many studies were able to demonstrate an ionophoric effect which is either directly linked to the membrane insertion of the toxin itself or represents a secondary effect of toxin action. Therefore, we examined the transcriptome profile of genes belonging to membrane-spanning ion transporters (like *ENA1*, *TRK1*, and *TRK2*) which could help the cell managing ionic disturbances. We solely found *PMA1*, the gene for the major plasma membrane H^+^-ATPase regulating cytoplasmic pH and plasma membrane potential, to be down-regulated over all time points; both *PMA1* regulators (*PMP1* and *PMP2*) were also down-regulated. Surprisingly, genes of the multiple drug transporters *PDR5* and *PDR12* belonging to the family of ABC-transporters were observed to be significantly down-regulated.

### De-Regulation of Transcription Factors

**FIGURE 4:**
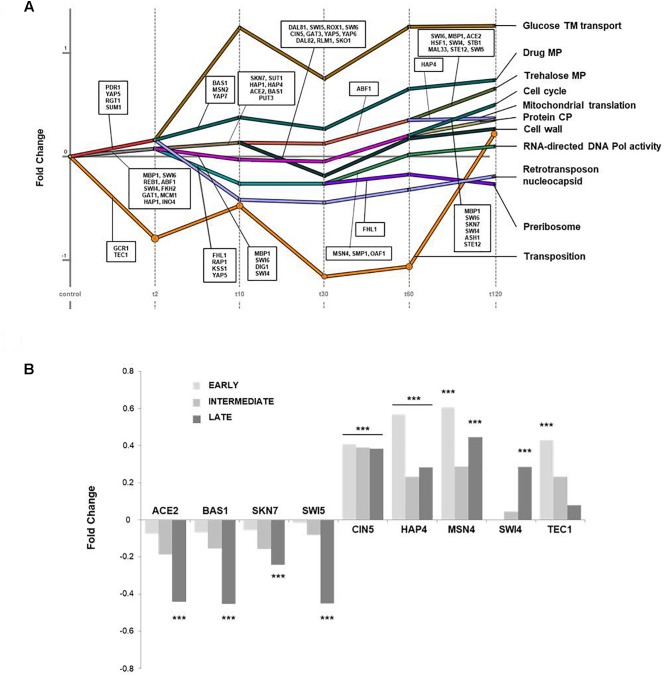
Analysis of the time-course experiment using DREM. The *Dynamic Regulatory Events Miner* (DREM) was used for clustering and visualizing the time-course expression dataset as a function of transcription factor-gene interactions. The TF assignment was limited to a threshold score of *p* < 0.01. **(A)** Dynamic regulatory map. Depicted is the dynamic regulatory network graph with the respective bifurcation events and the assigned TFs. The fold enrichment and the corresponding *p*-value of each GO term are illustrated in Table 1. **(B)** Significantly de-regulated TFs. The assigned TFs for each bifurcation were analyzed for transcriptome alterations over time. Depicted are the respective fold changes of significantly down-regulated (left) and up-regulated TFs (right) for each time cluster EARLY (light gray), INTERMEDIATE (gray), and LATE (dark gray). Significance is indicated with asterisks (^∗∗∗^ adjusted *p* < 0.01).

We used the ‘Dynamic Regulatory Events Miner’ (DREM) to obtain a clustering of genes according to time-course gene expression and TF-gene regulation (Schulz et al., 2012). This tool analyzes dynamic gene regulatory networks as a function of TF-gene interactions and generates a regulatory map in which the main bifurcation events in the given expression data and the potentially responsible transcription factors are visualized. Therefore, the mean TPM value of the triplicates per gene was calculated for each sampling point and used as an input matrix with a minimum absolute expression change of 0.3 resulting in a dynamic network graph with 11 main bifurcation events (Figure 4A). The GO annotations for genes of the generated paths are depicted in Table 1 with the corresponding corrected *p*-values (*p*-value_corr_) and the assigned TFs. Initially, three splits can be found in the expression dataset, namely ‘transposition’ (orange), ‘mitochondrial translation’ (gray), and ‘glucose transmembrane (TM) transport’ (red). The latter are compartmentalized further into three splits each whereas ‘glucose TM transport’ was not significantly enriched anymore after *t*_10_. The path ‘mitochondrial translation’ is split again after *t*_10_, additionally generating the path ‘cell wall’ (dark green) and again after *t*_60_ resulting in the branch ‘trehalose metabolic (MP) pathway’ (olive green). The transcriptome profile of all assigned TFs was analyzed and nine were found to be de-regulated. Among these, *ACE2*, *BAS1*, *SKN7*, and *SWI5* were significantly down-regulated at late sampling points (Figure 4B, left). In contrast, *CIN5* and *HAP4* were significantly up-regulated over all time points, and *MSN4* was positively regulated for early and late incubation times, whereas the transcriptional profile of *SWI4* and *TEC1* showed a significant up-regulation at late and early sampling points, respectively (Figure 4B, right).

**Table 1:** GO terms and assigned TFs identified by DREM.

Generic GO term	F.E.	*p*-value_corr._	Assigned TF (*p* < 0.01)
Glucose TM transport	3.9	2.0E-3	*PDR1, YAP5, RGT1, SUM1*
Glucose TM transport (after *t*_10_)	9.5	0.7	
Drug MP	2.3	3.4E-2	*BAS1, MSN2, YAP7*
Retrotransposon nucleocapsid	2.3	1.0	
Mitochondrial translation	5.6	1.5E-31	*MBP1, SWI6, REB1, ABF1, SWI4, FKH2, GAT, MCM1, HAP1, INO4*
Mitochondrial translation (*t*_2_–*t*_10_)	3.6	2.3E-21	*SKN7, SUT1, HAP1, HAP4, ACE2, BAS1, PUT3*
Mitochondrial translation (*t*_30_–*t*_120_)	5.6	1.5E-31	*ABF1, HAP4*
Trehalose MP	17.6	2.0E-3	*SWI6, MBP1, ACE2, HSF1, SWI4, STB1, MAL33, STE12, SWI5*
Cell wall	3.2	1.0	*DAL81, SWI5, ROX1*
Cell cycle	1.9	1.8E-11	*MBP1, SWI6, DIG1, SWI4*
Cell cycle (after *t*_60_)	3.2	1.7E-12	*MBP1, SWI6, SKN7, SWI4, ASH1, STE12*
Protein CP	2.4	6.0E-5	
RNA-directed DNA Pol activity (*t*_2_)	2.6	3.6E-17	*FHL1, RAP1, KSS1, YAP5*
RNA-directed DNA Pol activity (*t*_30_)	4.7	9.0E-3	*MSN4, SMP1, OAF1*
Preribosome	6.4	2.8E-32	*FHL1*
Transposition	22.4	3.7E-19	*GCR1, TEC1*


*The Dynamic Regulatory Events Miner (DREM) software was used for clustering and visualizing the time-course expression dataset and generic GO terms for each bifurcation event; the corresponding corrected p-value, and the fold enrichment (F. E.) are listed. The TF assignment was limited to a threshold score of p < 0.01 and the assigned TFs were depicted for the respective GO term. Each TF was examined for significant alteration and 9 were found to be de-regulated (see Figure 4B).*

### Gene Deletions Altering K1 Toxin Sensitivity

**FIGURE 5:**
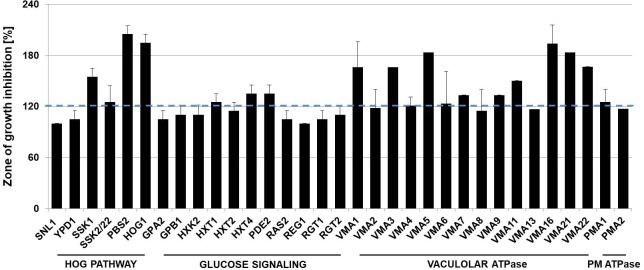
K1 toxin sensitivity of deletion mutants. Toxin sensitivity was analyzed via MBA by embedding 10^6^ cells of the respective mutant into the agar and applying 100 μl of K1 concentrate. The killing zone was measured after incubation of 3 days at 20°C, and normalized to the zone of growth inhibition of the wild-type strain BY4742 (100%). Hypersensitivity was defined as 120% and above, and is indicated by the dashed blue line (*n* = 5). PM: plasma membrane.

Extensive screening of *S. cerevisiae* mutants has already been performed highlighting many genes essential for toxin action and sensibility which could be non-randomly clustered according to their function. Especially genes affecting cell wall and mannoprotein synthesis, lipid and sterol biosynthesis, as well as secretory pathway trafficking, were found to contribute to the cellular reaction to K1 toxin (Page et al., 2003). We tested deletion mutants of components of the HOG pathway, the general glucose sensing machinery, and the multi-drug response. Additionally, mutants defective in the gene coding for the plasma membrane ATPase Pma1p, and its isoform Pma2p were analyzed as well as various genes encoding the subunits of the vacuolar ATPase (V-ATPase) to extend the initial screening. Consistent with the results of Page et al. (2003), we observed strong hypersensitive phenotypes for all kinase components of the HOG pathway with killing zone diameters twice that of the wild-type. In case of mutants defective in glucose signaling a tendency to a hypersensitive phenotype for the glucose transporters Hxt1p, Hxt2p, and Hxt4p could be monitored (Figure 5). The exposition of deletion mutants of the V-ATPase resulted in distinct hypersensitivity against the applied K1 toxin for the majority of the mutants. Because Pma1p is an essential gene, a diploid strain carrying one mutant and one wild-type allele was used in the analysis resulting in a light hypersensitivity against K1 toxin. In contrast, Δ*pma2* mutants showed no altered sensitivity (Figure 5).

### K1-Dependent Alterations of Intra- and Extracellular Metabolite Levels

**FIGURE 6:**
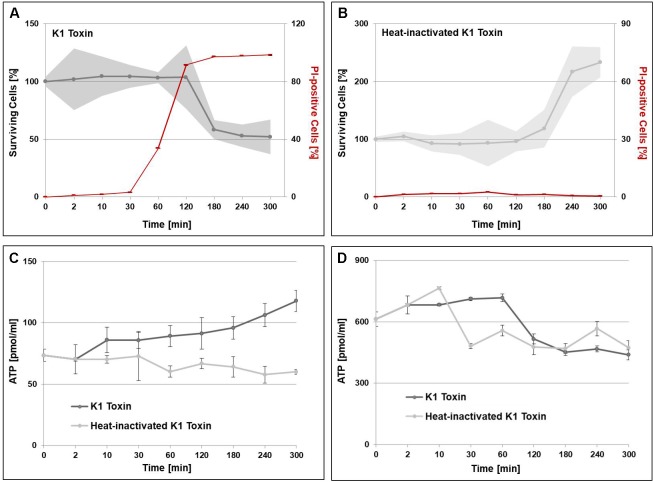
Time-resolved effects of K1 toxin application. BY4742 cells were cultivated in JG medium until OD_600_ 0.5 was reached and subsequently incubated with 1,000 AU K1 toxin concentrate, and samples were taken over the course of 5 h. Control samples were prepared by application of heat-inactivated K1 toxin. **(A,B)** Pore formation was determined by propidium iodide (PI) staining and subsequent FACS analysis with 100,000 gated events for each sample (*n* = 3). Samples for determination of cell viability were collected simultaneously, washed with 1 × PBS and plated on YPD agar plates considering appropriate dilutions. **(C,D)** Extraction of metabolites was performed by initially quenching the samples in 60% methanol (–40°C), collection of the supernatant (extracellular, **C**) and lysis of the remaining cell pellet with methanol-chloroform and glass beads (intracellular, **D**). ATP levels were determined via luciferase-activity by measuring the resulting luminescence over an integration time of 1,000 ms.

Qualitative PI-staining of K1-treated cells already showed a latency time of approximately 80 min until the first PI-positive cells can be detected. In a quantitative approach, we aimed to correlate the toxin-induced disruption of the plasma membrane resulting in a PI-positive staining with the decrease in cell viability. Therefore, BY4742 cells were incubated with either 1,000 AU K1 or heat-inactivated toxin and (Figure 6A,B). Interestingly, PI-positive cells can be observed over an hour before a significant lethal effect on the cells can be monitored pointing to an initial resistance against the membrane disruption. Control cells treated with heat-inactivated toxin showed no PI-positive cells verifying the impenetrability of the intact plasma membrane for this DNA-intercalating stain. Additionally, we determined the intracellular level of glycerol as well as the intra- and extracellular level of ATP. In case of the former, stable glycerol concentrations of 30–35 nmol/ml were detected with no significant alterations over time between the toxin-treated samples and the respective controls. Measurement of extracellular ATP concentrations clearly showed increased loss of the metabolite in toxin-treated cells with steady levels in the control samples (Figure 6C). Intracellular ATP of K1 toxin-treated cells remained stable until 120 min followed by a constant decrease of the metabolite. In contrast, cells treated with heat-inactivated toxin showed an initial decrease in intracellular ATP manifesting in stable levels of the metabolite over the remaining sampling points (Figure 6D).

## Discussion

**FIGURE 7:**
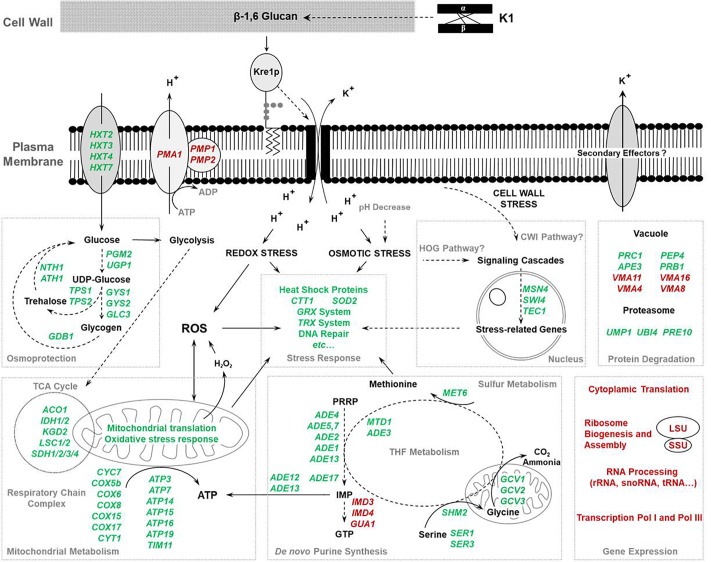
Transcriptional changes of a sensitive cell in response to killer toxin K1. After binding to the β-1,6-glucan fraction of the cell wall, K1 is transferred to the plasma membrane where it interacts with its secondary receptor Kre1p. Subsequently, insertion into the plasma membrane evokes a proton influx followed by potassium efflux. To date, possible interactions with secondary effectors contributing to the toxic effect cannot be excluded at this stage (Breinig et al., 2002). The uncontrolled accumulation of protons within the cytoplasm not only leads to a decrease of the intracellular pH but also to the induction of osmotic and oxidative stress responses cumulating in the up-regulation of several stress-responsive genes. Moreover, osmotic stress and disturbances in the cell wall maintenance induce HOG and CWI pathways, further adapting gene expression to stress defense. Besides systems for ROS detoxification, heat shock proteins, and DNA damage repair systems, the metabolism of the osmolytes trehalose and glycogen is positively regulated. Additionally, a remarkable increase in genes involved in TCA cycle, *de novo* purine synthesis, and THF metabolism takes place. Together with the sharp decrease in genes generally involved in gene expression and the down-regulation of genes encoding ATP-consuming proteins (*PMA1*, *VMA4*, *VMA8*, *VMA11*, and *VMA16*), K1 intoxication shifts the cellular metabolism to elevated ATP generation and saving. Green color: up-regulation; red color: down-regulation.

Microorganisms need to possess a broad variety of cellular mechanisms to resist several kinds of stress like heat, osmotic or oxidative stress. Therefore, rapid transcriptome responses to a changing environment are crucial for coping with these otherwise toxic alterations. For the first time, we analyzed the transcriptional profile changes of *S. cerevisiae* in response to application of the ionophoric K1 toxin in a time-series experiment. Our results reveal the ability of the viral killer toxin K1 to trigger massive transcriptome alterations in a sensitive yeast (summarized in Figure 7).

### Down-Regulation of RNA Metabolism and Ribosomal Assembly

Our results showed a rapid decrease in genes coding for both ribosomal SU as well as genes mediating RNA processing and subunit assembly. The down-regulation of ribosome-associated genes observed already at very early stages after K1 application reflects a typical cellular reaction to different environmental stress situations (Warner, 1999; Gasch et al., 2000). Genes of mitochondrial ribosomal proteins were instead up-regulated which is in clear contrast to the moderate transcriptional down-regulation of this gene category after treatment with *Pichia membranifaciens* killer toxin PMKT (Santos et al., 2005). The negative regulation of ribosomal components was stable over time indicating a down-regulation of general gene expression and protein synthesis, presumably in favor of increased expression of protective proteins (Jelinsky and Samson, 1999). Furthermore, it might hint at an overall reduction of ATP consumption as ribosome synthesis and assembly are known to require a considerable amount of energy (Kressler et al., 2010).

### Induction of Stress Responses

Application of K1 toxin to sensitive yeast cells induced the de-regulation of various genes encoding proteins connected to general stress and osmotic shock response, and especially processes associated with oxidative stress responses. In yeast, stress responses can be modulated by the regulation of two pathways referred to as high osmolarity glycerol (HOG) and cell wall integrity (CWI) pathway (Qi and Elion, 2005). The CWI pathway is primarily induced by cell wall stress cumulating in the activation of the MAPK Stl2p (Levin, 2005). The highly conserved HOG pathway represents the central signaling pathway for modulation of osmotic stress responses leading to the activation of the MAPK Hog1p. After dual phosphorylation of Hog1p by the MAPKK Pbs2p, the protein modulates various cytoplasmic and nuclear responses in order to adapt the cellular metabolism to osmotic stress (Ferrigno et al., 1998; Reiser et al., 1999). Except for Stl2p, we found no significant up- or down-regulation of the participating kinases of the HOG and CWI pathway, respectively. Taken into account that MAPK signal cascades are mostly regulated by phosphorylation events, this lack of transcriptional de-regulation is not surprising. However, we found a significant up-regulation of *MSN4* (early and late) and also *SWI4* (late) upon K1 intoxication. Msn4p and its paralog Msn2p (Msn2/4p) are rapidly displaced to the nucleus upon phosphorylation via the HOG pathway where they bind to conserved stress-response elements (STRE) found in many stress-responsive genes (Martinez-Pastor et al., 1996; Schmitt and McEntee, 1996; Gorner et al., 1998). Swi4p is a transcriptional activator regulating the transcription of G1-specific genes and also genes essential for DNA repair mechanisms upon activation by kinases of the CWI pathway (Workman et al., 2006). Further, our transcriptional analysis revealed the up-regulation of many Msn2/4p and Swi4p target genes (Zhou and Elledge, 1993; Rep et al., 1999; Amoros and Estruch, 2001). Taken together, K1 intoxication presumably led to the activation of both signaling pathways cumulating in the expression of various stress-responsive genes. Toxin sensitivity of mutants defective in different HOG pathway components were analyzed via MBA assay, and a distinct hypersensitive phenotype was observed confirming prior results of a genome-wide mutant screening. However, in this screen, deletion mutants defective in CWI pathway components were found to display a wild-type phenotype (Page et al., 2003). Interestingly, a genomic screen of yeast cells incubated with K2 killer toxin could show a hypersensitive phenotype of Δ*hog1* and various mutants of the CWI pathway (Servienė et al., 2012). Taken together, this illustrates the general importance of the HOG pathway in cellular stress responses whereas the CWI pathway seems to play a more crucial role in K2 toxicity. To further investigate the relevance of intracellular signaling cascades in K1 toxin action, the activation of both participating kinases and the involved TFs has to be further analyzed.

Just after toxin addition, we found stress-responsive genes up-regulated linked to oxidative stress including *SOD2* and *TSA1*. This observation is further supported by the positive transcriptional regulation of *GRX1*, *GRX2*, *GTT1*, *HYR1* (*GPX3*), *PRX1*, *TRX1*, and *CTT1* as intermediate and late responding genes, hinting at a continuous increase of the oxidative stress level. Elevated levels of reactive oxygen species (ROS) can damage cellular structures, like proteins, lipids, and DNA, and dysbalance the reductive capacity of the yeast cytoplasm. Therefore, yeast cells possess conserved detoxification systems to survive toxic amounts of ROS, involving catalases, superoxide dismutases, and peroxidases. The most prominent peroxidases in yeast are glutathione (GPXs) and thioredoxin peroxidases (TRXs) playing crucial roles in cellular defense mechanisms against oxidative agents. In *S. cerevisiae*, three GPX-coding genes are described with HYR1 (GPX3) encoding the most important protein acting as a signal transducer for the TF Yap1p, the major regulator of oxidative stress-related genes, and additionally accounting for the majority of the glutathione peroxidase activity (Inoue et al., 1999; Avery and Avery, 2001; Grant, 2001; Delaunay et al., 2002). The cytoplasmatic TRX peroxidase Tsa1p protects thiol-containing oxidation systems and can remove hydrogen peroxide and alkyl hydroperoxides in interaction with the thioredoxin system. Additional studies demonstrated a chaperone-like function of the protein besides its peroxidase activity (Park et al., 2000; Jang et al., 2004). Moreover, cells have to handle already damaged cellular parts. In this respect, proteins containing amino acids with sulfur residues are often a target for oxidative damage and have to be repaired by specific cellular mechanisms. Among these, glutaredoxin (GRX) and the thioredoxin (TRX) systems are the most prominent ones. The yeast cytosol contains two glutathione (GSH)-dependent glutaredoxins encoded by *GRX1* and *GRX2* and the thioredoxins Trx1p and Trx2p. Taken together, as a response to K1 toxin, our analysis revealed numerous parts of the defense system against oxidative stress localized in the mitochondria as well as in the cytosol. Moreover, the gradual increase of up-regulated genes from early to late sampling points toward a continuous rise of oxidative damage in the cell, also explaining the up-regulation of other stress-related genes. Notably, the positive upregulation of *XBP1*, *MSH1*, *MSH2*, and *DDR2* might point to an increase in cellular DNA damage at late stages of killer toxin action.

### Regulation of Transcription Factors

Besides the already mentioned TFs Msn4p and Swi4p, seven transcriptional regulators identified via DREM analysis were significantly altered. *ACE2*, *SKN7*, *SWI5*, and *BAS1* showed a down-regulation at late sampling points, *CIN5*, *TEC1*, and *HAP4* were up-regulated. The TF Bas1p partly controls the transcriptional regulation of purine biosynthetic reactions and is involved in the modulation of the THF pathway (Denis and Daignan-Fornier, 1998; Denis et al., 1998). *SKN7*, in contrast, is essential for osmoregulation and an oxidative stress-related induction of different heat-shock genes. *ACE2* and its paralog *SWI5* are involved in regulation of cell cycle events, cell morphogenesis, and mating type switches whose down-regulation after prolonged incubation with K1 underlines the increase in DNA stress and a stop in cell division (Sbia et al., 2008). Additionally, *XBP1* was also up-regulated at late sampling points, which plays an essential role in DNA replication and ER stress. Cin5p as part of the Yap1p family is involved in stress response by mediating pleiotropic drug resistance (Mendizabal et al., 1998; Furuchi et al., 2001). The TF Hap4p, in contrast, represents a transcriptional activator and is as part of the Hap2/3/4/5p CCAAT-binding complex involved in respiration-related gene regulation and diauxic shift (Forsburg and Guarente, 1989; Zampar et al., 2013). Due to the lacking transcriptional alteration of genes encoding the remaining complex subunits, we suppose that the overall up-regulation of *HAP4* might reflect a fine-tuning of the whole CCAAT-binding complex. Further, *HAP4* plays a role in mitochondrial biogenesis, respiration, and osmotolerance, evoking a higher stress resistance, an increase in cellular metabolism, and an extended lifespan (Lascaris et al., 2003; Shi et al., 2018). Finally, Tec1p is involved in expression of Ty1 retrotransposable elements. Yeast possesses five types of retrotransposons named Ty1-5 which are parasitic elements integrated into the host genome (Kim et al., 1998). It has been shown that environmental signals can regulate the transcriptional level of Ty elements thereby activating and repressing the transcription of retrotransposons (Lesage and Todeschini, 2005; Beauregard et al., 2008) explaining the distinct up- and down-regulation of Ty-retrotransposons observed after application of K1.

### Adaptions in Energy Metabolism

We also found several carbohydrate metabolism-related genes starting directly at brief incubation times (10 min) and staying consistently up-regulated over all time points. When preferred sugars are diminished, yeast cells rapidly adapt their whole metabolism. Although nutrient stress is diversely defined, recent studies on glucose-dependent cytoplasmic pH adjustment showed striking starvation effects at glucose levels below 11 mM (Isom et al., 2018). Taken this into account and to avoid a bias of the data in our experimental setup, we adjusted the resulting glucose concentration after addition of the toxin concentrate considerably above this critical concentration (37 mM). Hence, the observed up-regulation of glucose transporters except for the low-affinity transporter Hxt1p is not based on glucose starvation but a consequence of osmotic stress as it has been shown for the up-regulation of various carbohydrate metabolism-related genes (Gasch et al., 2000; Rep et al., 2000). Interference of the glucose transport due to disturbance of membrane integrity induced by K1 could result in, e.g., an efficiency loss in glucose uptake and malfunctions of the respective transporters. However, further experimental data are necessary to validate the importance of these transporters in K1 toxin action.

Although only a few genes involved in glycolysis were found to be altered by K1 application, we found almost the complete set of genes encoding enzymes of the TCA cycle positively regulated. Moreover, up-regulation of the cytochrome c isoform *CYC7*, as well as *COX15* and *COQ5*, both necessary for cytochrome c oxidase biosynthesis and assembly, could promote ATP generation through increased respiratory capacity. However, under physiological conditions, the glycolytic flux is controlled in highly complex mechanisms including several feed-back loops according to the needs of the cell and extracellular supplies. Although cells can adjust the physiological state to environmental conditions and K1 intoxication could interfere with the glycolytic control mechanism, the observed de-regulation of genes essential for respiration has to be verified on protein level. In a first attempt, we analyzed the K1 sensitivity of different deletion mutants defective in glucose sensing genes. Although, a tendency to a hypersensitive phenotype in case of Δ*hxt1*, Δ*hxt2*, Δ*hxt4*, and Δ*pde2* was observed, most of the mutants showed a wild-type reaction to K1 application. Especially the phenotype of Δ*pde2* can be explained by the decrease of the cytosolic pH which could enforce and accelerate K1 toxicity (Isom et al., 2018).

Furthermore, our data prove the positive transcriptional regulation of many genes related to purine *de novo* biosynthesis and tetrahydrofolate (THF) metabolism. After the generation of several intermediates, inosine monophosphate (IMP) is formed by the gene products of *ADE16* and *ADE17* (Tibbetts and Appling, 2000). IMP acts as a precursor molecule for either the formation of GTP via Imd3p, Imd4p, and Gua1p or the generation of ATP. Interestingly, and in clear contrast to the up-regulation of many *ADE* genes, we observed a significant down-regulation of *IMD3*, *IMD4*, and *GUA1* transcripts indicating a shift of the purine biosynthesis pathway to the generation of ATP. In line with this, genes directly involved in the cycling of THF were also positively regulated. Summarized in the GO term ‘one carbon metabolism,’ the respective gene products are involved in serine synthesis (*SER1* and *SER3*), serine to glycine conversion (*SHM2*) and subsequently glycine degradation (*GCV1*, *GCV2*, and *GCV3*) and play a crucial role in THF metabolism by generating and consuming THF derivatives (Piper et al., 2000).

Increase in respiratory capacity and, as a consequence, a higher generation of ROS explains the up-regulation of oxidative stress-defensive constituents in both mitochondria and cytoplasm. Dysfunction in mitochondria can induce several different signal cascades altering the cellular metabolism being especially caused by incorrect assembly of the respiratory chain complex. Although the complete mechanisms of signal transduction are yet unclear, alterations in mitochondrial peptides released into the cytoplasm can induce the down-regulation of cytoplasmic translation with simultaneous up-regulation of mitochondrial translation as observed in this study (Arnold et al., 2006; Suhm and Ott, 2017). Moreover, it has been shown that disturbances in mitochondrial protein import can lead to accumulation of precursor proteins accompanied by induction of proteostasis and a stop in cytosolic translation to regulate protein homeostasis (Wrobel et al., 2015). Additionally, genes of the vacuolar degradation machinery (*PRC1*, *PEP4*, *PRB1*, and *APE3*) and the proteasome were found to be up-regulated pointing to elevated protein degradation, probably to increase nutrient availability during stress situations.

We also found genes for ATP-consuming and binding proteins to be significantly down-regulated within the first 10 min hinting at a reduction of ATP consumption in the early phase after K1 application. In intermediate and late stages, a significant de-regulation of these genes could no longer be detected, possibly due to increased demands for heat shock proteins; this would be in line with the up-regulation of various genes of this family at late stages of K1 intoxication (e.g., *HSP12*, *HSP26*, and *HSP42*). Interestingly, we observed a consistent down-regulation of *PMA1* and also *PMP1* and *PMP2* encoding Pma1p-regulating proteins over time. This might reflect a secondary effect presumably caused by the decreasing intracellular pH due to toxin action. As the main proton pump of the cytoplasmic membrane, Pma1p represents one of the major ATP consumers and is essential for cytosolic pH regulation and the electrochemical proton gradient (Serrano et al., 1986; Rao et al., 1992; Morsomme et al., 2000). This P-type ATPase is one of the most abundant and stable plasma membrane proteins with a half-life of approximately 11 h (Benito et al., 1991; Morsomme et al., 2000). In addition to the severely reduced activity of the plasma membrane ATPase in *pmp1-pmp2* mutants, internalization of Pma1p could be shown as a consequence of deletion or inhibition of the vacuolar ATPase (V-ATPase) (Navarre et al., 1994; Martinez-Munoz and Kane, 2008). This multi-subunit proton pump is responsible for the acidification of vacuolar, endosomal and lysosomal compartments. We observed the down-regulation of genes coding for subunits of the V_0_ domain (*VMA11* and *VMA16*) and V_1_ domain (*VMA4* and *VMA8*), respectively. Interestingly, a screening of mutants defective in V-ATPase subunits as well as *PMA1* showed a distinct hypersensitive phenotype with killing zones comparable to Δ*hog1* mutations. This phenomenon is most probably linked to a disturbance in pH homeostasis cumulating in accumulation of H^+^ ions in the cytoplasm. This acidification of the cytosol and the defective vacuolar transport of protons could maximize the toxic effect of K1. Therefore, the actual protein level and the localization of both ATPases, as well as the remaining activity of Pma1p, should be investigated in the near future.

The increase in intracellular ATP suggested by our data indicates an elevated energy consumption of the sensitive cell. In line with this, studies on mammalian cells incubated with the bacterial pore-forming toxins Aerolysin and Listeriolysin O, which resemble the potassium efflux of K1, were able to show an ATP-dependent regeneration of plasma membrane integrity and ionic balance. This repair mechanism correlates with stability, dimension, and abundance of the pores (Gonzalez et al., 2011). Likewise, the increased ATP requirement might be a consequence of a comparable repair mechanism in sensitive yeast. This hypothesis is supported by the observed delay of measurable cell death compared to the appearance of first PI-positive cells indicating the disruption of the plasma membrane. However, the loss of different metabolites including ATP indicates that sensitive cells are presumably not able to disintegrate the toxin pores or fail in repairing the damaged membrane after prolonged intoxication (Bussey and Sherman, 1973). Alternatively, the observed shift toward ATP formation might reflect the recently described hydrotropic function of ATP, thus, maintaining protein solubility independent of chaperones (Patel et al., 2017). Determination of extra- and intracellular ATP levels verified the K1-induced loss of the metabolite due to disruption of plasma membrane integrity already described in early studies (Bussey and Sherman, 1973). Interestingly, intracellular levels remained stable until 120 min after toxin application although the increase in extracellular ATP was observed significantly earlier. This observation strengthens the results obtained by the transcriptome analysis as a metabolic shift toward ATP production seems to stabilize intracellular ATP content.

### Regulation of Osmolyte Metabolism

The accumulation of osmolytes like trehalose, glycogen, and glycerol has already been shown to be a critical cellular defense mechanism against various stress situations, helping in detoxification and stabilization of cellular proteins (Yancey, 2005). Accordingly, every GO enrichment performed pointed to the importance of genes associated with trehalose and glycogen metabolism. We found the majority of genes related to the biosynthesis of these osmolytes up-regulated after 10 min with stable high expression levels overall. This molecule serves not only as an energy supply but also directly as osmoprotectant by stabilizing misfolded proteins and buffering desiccation events; additionally, an involvement in ROS detoxification and reduction of lipid peroxidation was proposed (Allison et al., 1999; Benaroudj et al., 2001; Pereira Ede et al., 2003; Kandror et al., 2004; da Costa Morato Nery et al., 2008). It has also been shown that genes encoding for glycogen and trehalose biosynthesis and their common precursor UDP-glucose are stress-controlled (Winderickx et al., 1996; Parrou et al., 1997; Parrou et al., 1999). Interestingly, in addition to genes connected to trehalose and glycogen biosynthesis up-regulated directly at early stages of K1 toxin application, we also found *NTH1*, *NTH2*, and *ATH1* as well as *GPH1* and *GDB1* positively de-regulated at late sampling points encoding enzymes for trehalose and glycogen degradation. This futile cycling, observable under diverse stress conditions (Parrou et al., 1997, 1999), needs considerable amounts of energy and might, thus, contribute to ATP consumption. Additionally, it has been shown that trehalose can stabilize the membrane structure by being transported to the outside of the plasma membrane (da Costa Morato Nery et al., 2008).

Another critical osmolyte is glycerol, a simply structured polyol rapidly accumulated upon cellular stress (Klipp et al., 2005). It is produced by reduction of dihydroxyacetone-phosphate to glycerol-3-phosphate that is subsequently dephosphorylated. The first step is catalyzed by NAD-dependent glycerol-3-phosphate dehydrogenase, encoded by *GPD1* and its isoform *GPD2*, whereas dephosphorylation is conducted by the gene products of *GPP1* and GPP2, two paralogs coding for DL-glycerol-3-phosphate phosphatase. Surprisingly, we observed a sharp decrease in the transcripts of most glycerol-related genes. Immediately after K1 application, *GPD1* and *GPP1* were down-regulated followed by *GPD2* and *GPP2* at intermediate time points. This observation is in clear contrast to comparable transcriptome profiling experiments of yeast cells incubated with the *P. membranifaciens* killer toxin PMKT yielding a strong up-regulation of *GPD1* and *GPP2* (Santos et al., 2005). As the sensitive strain in our experiment was metabolically pre-adapted to glycerol-containing medium, this observation might suggest that glycerol molecules might somehow pass the plasma membrane after toxin application evoking a negative feedback regulation on the respective genes. Although K1-formed channels in artificial lipid bilayers were shown to be cation-specific, potential passive diffusion of non-charged molecules cannot be entirely excluded (Martinac et al., 1990). Alternatively, glycerol influx might occur as a side effect of toxin action, supposedly by disturbing the open-close-state of the glycerol transporter Fps1p. The increase in intracellular glycerol concentration would thereby explain the down-regulation of glycerol synthesis-related genes as well as the up-regulation of genes involved in glycerol utilization like *GUT2*. Measurement of intracellular glycerol levels showed no significant alterations indeed in the concentration of this metabolite neither over time nor when comparing toxin-treated cells with control samples. This contradicts a potential accumulation over time but could hint to an equilibrated glycerol flux over the plasma membrane through K1-induced pores and the glycerol transporter Fps1p. Additionally, the analysis was hindered by the presence of relatively high concentrations of free extracellular glycerol in the extracellular medium, and the experiments should be repeated using glycerol-free growth medium to minimize potential artifacts. However, a genome-wide screen conducted on mutants of *S. cerevisiae* showed wild-type phenotypic reaction to extracellular K1 application for a Δ*gpd1-gpd2* double deletion mutant, arguing against a critical role of the compatible osmolyte glycerol in K1 resistance (Page et al., 2003).

### Metabolic Changes as Defense Mechanism Against Ionophoric Toxins

By disrupting the plasma membrane gradient, K1 drains the intoxicated target cell energetically and electrochemically. Different studies implied a lag phase after toxin application as the first dead cells were detected only after approximately 1 h. Interestingly, this period between toxin application and first detection of PI-positive cells is not unique for K1 but has also been observed for the *P. membranifaciens* PMKT toxin that resembles K1 in certain features (Santos et al., 2005). This remarkable similarity could point to a general mechanism initializing or preceding the toxic effect of yeast-derived ionophoric killer toxins. Excluding the rather fast binding of the toxin to the glucan fraction of the cell wall and its rapid transfer to the plasma membrane, this lag period might include several events concerning either pore-formation by potential oligomerization of K1 monomers and/or metabolic and structural changes as defensive reaction of the sensitive cell (Skipper and Bussey, 1977; Bussey, 1991; Kurzweilova and Sigler, 1995). Our approach yielded various novel insights in these so far hypothetical metabolic adaptations reaching beyond the classically described effects of ionophoric toxins and explains the observed lag phase, thereby, adding a new facet to K1 biology. A possible impact of the genes described in this study will be further investigated by testing respective mutants against externally applied toxin and the recently constructed intracellular K1 derivatives as the analysis of several yeast deletion mutants is hampered by the fact that respective genes are involved in or at least have a significant influence on presence and/or amount of the toxin’s cell wall receptor β-1,6-glucan (Gier et al., 2017). These experiments will help to clarify the molecular events underlying K1 toxicity and immunity and might lead to novel insights into the metabolic effects of medically relevant ionophoric toxins using the eukaryotic model organism *S. cerevisiae*.

## Author Contributions

FB, SG, MS, and MJS conceived and designed the experiments. SG conducted K1 treatment and RNA isolation. KN and MS performed Illumina sequencing. MHS, SK, and SG analyzed transcriptome data. SG and FB interpreted the results and wrote the manuscript.

## Conflict of Interest Statement

The authors declare that the research was conducted in the absence of any commercial or financial relationships that could be construed as a potential conflict of interest.

## Footnotes

1 http://www.ebi.ac.uk/ena/data/view/PRJEB28672

## Supplementary Material

Table S1 Number of Reads. This list includes the number of reads obtained by Illumina HiSeq2500 platform sequencing, and the number of reads used for following quantification via Salmon software. Read length after adaptor trimming constituted of 20–50 nt.

Table S2 Complete list of significantly altered DEGs after K1 application. ORFs were clustered into up- or down-regulated DEGs according to their log2 fold change (UP ORF and DOWN ORF, respectively). Enumerated in alphabetic order are all ORFs showing a significant de-regulation (adjusted *p* < 0.01) at one time cluster (early, intermediate, and late) minimum. Significance is indicated by coloration (green: up-regulated, Red: down-regulated, gray: not significant) as well as the corresponding log2 fold change; six genes showed fluctuating fold changes over time and are classified separately (FLUCTUATING ORF).

Table S3 Complete list of globally enriched GO terms. GO enrichment for ‘Biological Process’ (BP), ‘Molecular Function’ (MF), and ‘Cellular Component’ (CC) was performed using the ‘Gene Ontology enRIchment anaLysis and visuaLizAtion tool’ (GOrilla) after initially clustering the significantly altered DEGs into up- and down-regulated ORFs (outlined as UP and DOWN). For each GO enrichment the respective GO terms, the *p*-value and its false discovery rate (FDR)-corrected *q*-value as well as the assigned DEGs (Genes) are depicted. *N*, total number of genes; B, genes associated with specific GO term; n, number of genes inserted; b, intersection. Enrichment is automatically calculated as (b/n)/(B/N).
